# PDBsum*1* : A standalone program for generating PDBsum analyses

**DOI:** 10.1002/pro.4473

**Published:** 2022-12

**Authors:** Roman A. Laskowski

**Affiliations:** ^1^ European Molecular Biology Laboratory European Bioinformatics Institute (EMBL‐EBI) Cambridge

**Keywords:** 3D protein structure, PDB, PDBsum, protein database, protein structure analysis, schematic diagrams

## Abstract

PDBsum1 is a standalone set of programs to perform the same structural analyses as provided by the PDBsum web server (https://www.ebi.ac.uk/pdbsum). The server has pages for every entry in the Protein Data Bank (PDB) and can also process user‐uploaded PDB files, returning a password‐protected set of pages that are retained for around 3 months. The standalone version described here allows for in‐house processing and indefinite retention of the results. All data files and images are pre‐generated, rather than on‐the‐fly as in the web version, so can be easily accessed. The program runs on Linux, Windows, and mac operating systems and is freely available for academic use at https://www.ebi.ac.uk/thornton-srv/software/PDBsum1.

## 1 INTRODUCTION

PDBsum is a web server developed at University College London (UCL) in 1995^1^ and moved to EMBL‐EBI in 2002, where it currently resides. From the start, its aim was to provide a convenient source of information on every structure in the Protein Data Bank (PDB).^2^ The information is presented mainly in schematic diagrams across several pages relating to the different components of the structure—protein, ligands, DNA, and the interactions between them. The server is updated weekly and cross‐referenced to related servers such as Sequences Annotated by Structure (SAS),^3^ ProFunc,^4^ and VarSite.^5^ Recent additions have included analyses of all experimentally determined protein structures of the SARS‐CoV‐2 virus, and all AlphaFold2 models of human proteins.^6^

As well as the above, PDBsum can also analyse user‐uploaded structures and return a password‐protected set of analyses for each. This feature is accessed via the ‘PDBsum Generate’ link on the home page (https://www.ebi.ac.uk/pdbsum). Once the calculations are complete, a link to the results, with password, is e‐mailed to the user. The turnaround time varies from a few minutes to several hours, depending on the complexity of the structure submitted and of any in the queue ahead of it. Currently, the server receives an average of 140 structures per day, or over 4,000 per month. For space reasons, the data can only be kept for around 3 months and, even though the results are password protected, some users may have concerns about their data being on a public server. For these reasons, a standalone subset of the PDBsum programs, called PDBsum*1*, has been developed for download and in‐house use. It can run on Linux, Windows, and mac operating systems. Some third‐party applications are required, as described below, but it is expected most users will already have these installed on their systems.

## 2 THE PROGRAMS

PDBsum*1* can be downloaded as a set of executables. The source code is also provided in case the executables prove to be incompatible with the user's operating system; recompiling the source code should allow the programs to run. The programs comprise a collection of C and FORTRAN programs, some of which date back to the early 1990s. They include the following.

- HBPLUS^7^—calculation of hydrogen bonds and non‐bonded contacts, as used by LIGPLOT and NUCPLOT.
- LIGPLOT^8^—schematic diagrams of protein‐ligand and protein‐metal interactions.
- NUCPLOT^9^—protein–DNA interaction diagrams.
- PROCHECK^10^—analyses of structural quality.
- ProMotif^11^—plots and tables describing various secondary structure motifs in the protein(s)—such as beta turns, helix–helix interactions, beta‐alpha‐beta units, and so forth.
- SURFNET^12^—calculation of cavities and surface clefts, using the updated algorithm called speedfill.

The controlling program, pdbsum1, runs each of the above programs in turn before generating a set of HTML pages and images presenting the final results. Also generated are RasMol^13^ and PyMOL^14^ scripts for viewing some of the analyses in these 3D molecular graphics viewers. Additional HTML pages provide indexes of all previous runs so you can keep track of all structures processed.

The program can be run on a single PDB entry, or on a number of entries listed in a text file. Each entry is identified by a four‐character ‘PDB code’. This will be extracted from the file name if it is of the form pdbXXXX.ent, where XXXX will be taken as the code. Alternatively, the program can use any four‐character code supplied at run‐time, or can automatically generate one—starting from a001, a002, and so forth.

## 3 RESULTS

**FIGURE 1:**
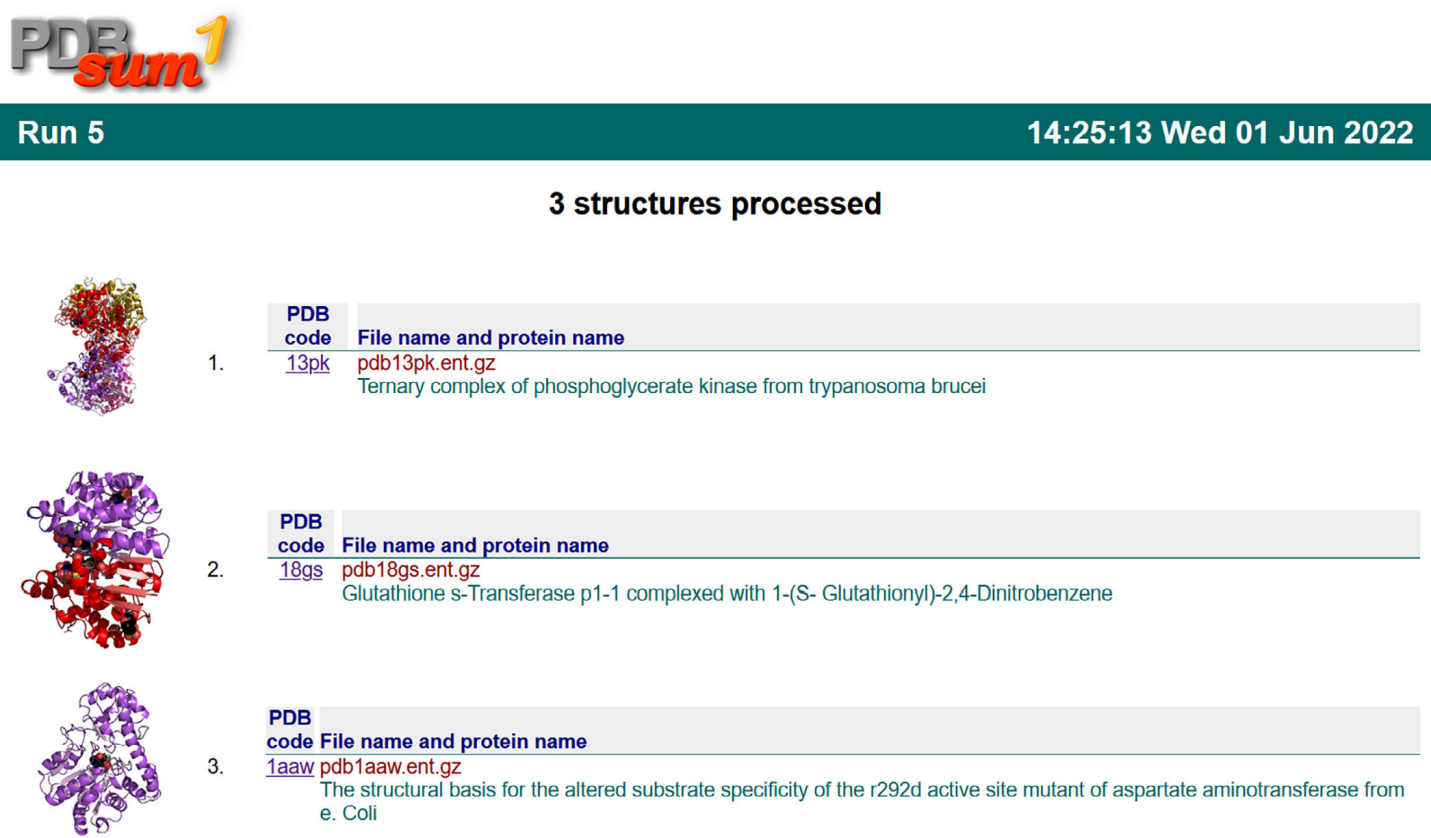
Example of an index.html file generated by a run of PDBsum*1*, listing the structures that were processed (three in this case). Clicking on either the thumbnail image or the PDB code takes you to the relevant results page (e.g., Figure 2a). Clicking on the PDBsum*1* logo at the top goes to a list of all prior runs

Figure 1 shows the index HTML page that lists the structures processed in the current run. Clicking on any structure goes to that structure's PDBsum*1* page. Prior runs can be accessed by clicking on the logo at the top of the page.

**FIGURE 2:**
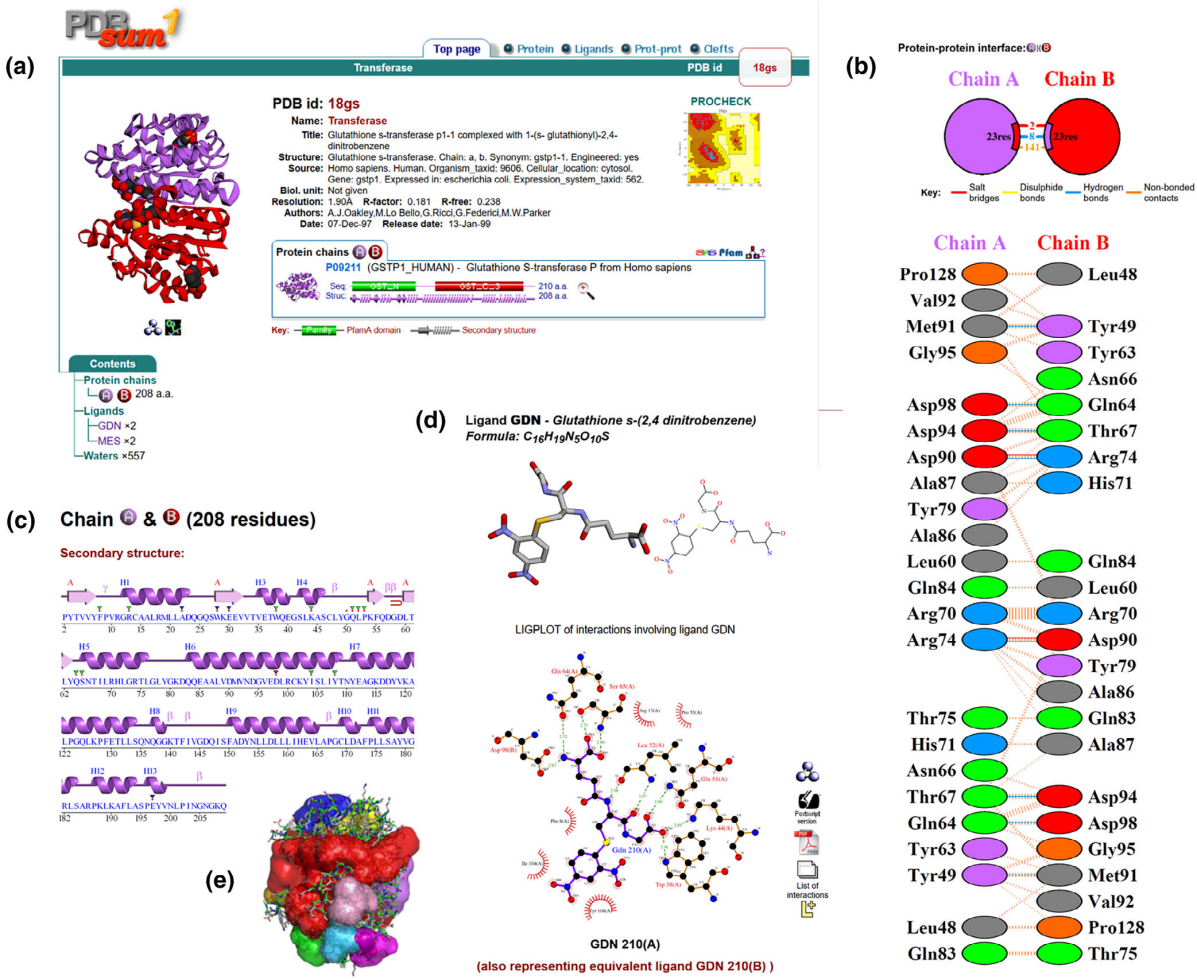
Extracts from the PDBsum*1* pages for PDB entry 18gs.^15^ (a) Main page showing an interactive 3D thumbnail image of the structure, a list of the file's contents in terms of proteins, ligands, metals. To the right is text information extracted from the file's header records. The domain diagram below the header information shows the protein's Pfam domains (green and red cylinders) and how much of the protein's 210 residues are covered by the 3D structure (purple strands and helices, 208 residues). (b) Interactions across the protein–protein interface between chain A (purple) and B (red) in terms of salt bridges (red lines), hydrogen bonds (blue lines), and non‐bonded contacts (orange lines). The coloured wedges in the top diagram represent the relative surface area of each chain involved in the interaction. The lower diagram shows the interacting residues on either side of the interface. (c) Schematic diagram showing the protein's sequence and corresponding secondary structure. Alpha helices are represented by the helical regions (labelled H1, H2, etc.), and beta strands as arrows (labelled A to indicate which beta sheet they belong to—in this case, there is only one). Also shown are beta and gamma turns, active site residues (coloured triangles), and residues interacting with the ligand (red dots). (d) Representations of the bound ligand (GDN) and the interactions it makes with surrounding protein residues. The top left view can be dynamically rotated and zoomed in and out with the mouse. The LIGPLOT diagram at the bottom shows a flattened schematic diagram of the interactions the ligand makes with the protein. Hydrogen bonds are shown as green dotted lines, while residues involved in non‐bonded contacts to the ligand are depicted by the orange eyelashes. The icons to the right of the diagram provide PostScript and PDF versions of the plot, as well as a simple list of the interactions, and a RasMol view of the interactions in 3D. If you have LigPlot^+^ installed, the L^+^ icon will load the plot into the program for editing and printing. (e) A representation of the clefts in the surface of the protein, coloured according to the volume of the cleft (with red as the largest). The clefts can also be viewed in RasMol and PyMOL

Figure 2 shows some of the PDBsum*1* outputs for PDB entry 18gs.^15^ The thumbnail image of the structure on the first page (Figure 2a) is a dynamic one, created using the JavaScript plug‐in, 3Dmol.js.^16^ Using the mouse you can rotate the image, or zoom in and out of it. The two icons beneath the thumbnail link to RasMol and PyMOL which, if your browser has been correctly configured, will display the structure in the respective molecular graphics viewer. The other displays in Figure 2 represent residue interactions across the dimer interface (Figure 2b), the protein's secondary structure (Figure 2c), a LIGPLOT diagram of the protein–ligand interactions (Figure 2d), and the different clefts in the protein's surface (Figure 2e).

**FIGURE 3:**
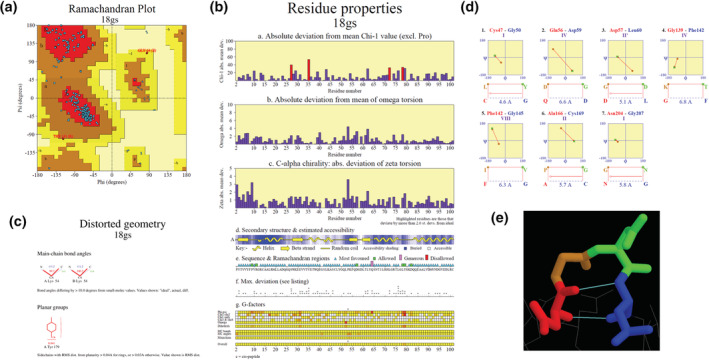
Examples of PROCHECK (a–c) and ProMotif (d–e) analyses for PDB entry 18gs. (a) Ramachandran plot of the mainchain φ‐ψ torsion angles, showing how the individual residues' values (blue squares) are distributed with reference to the most favoured (red), additional allowed (brown), generously allowed (dark yellow), and disallowed (light yellow) regions. Residues in the disallowed regions are represented by red squares and labelled. (b) Residue properties plot showing, for each residue, its absolute deviation from the mean χ_1_ value (box a), absolute deviation from mean ω torsion angle (box b), absolute deviation of the ζ torsion angle (box c), schematic representation of the secondary structure (like in Figure 1c), and average estimated accessibility, where the darker the blue the more buried the residue (Graph d), markers indicating which Ramachandran plot region (as in a), each residue falls in (Graph e), maximum deviation from ‘normality’ (Graph f), as taken from the residue‐by‐residue listing (not shown), and the various G‐factors (Graph g), as described in the PROCHECK documentation. (c) Distorted geometry plot showing two residues (Lys54 in chains A and B) with mainchain bond angles outside the norm, and one planar residue (Tyr179) which is not truly planar. (d) Beta turns in the structure, as computed by ProMotif. The plots show the four residues involved in each turn and where the φ‐ψ torsion angles of the central two residues lie on the Ramachandran plot. The class each turn belongs to (i.e., I, II, II′, etc.) is shown in purple. (e) A RasMol view of the third beta turn in (d) (Asp57‐Leu60), with the residues coloured according to their position in the turn: red (Asp57), brown (Gly58), green (Asp 59), and blue (Leu60). Hydrogen bonds are shown by the thin cyan lines

In Figure 3 are shown some examples of the outputs from the PROCHECK and ProMotif programs. The former presents a number of analyses of stereochemical features associated with the quality of a protein structure, including a Ramachandran plot, plots of χ_1_ and χ_2_ torsion angles, and diagrams showing any bonds, angles, or planar groups having distorted geometry. The ProMotif outputs include analyses of β‐ and γ‐turns, β‐hairpins, β‐bulges, β‐α‐β units, ψ‐loops, helix–helix interactions, as well as Hera^17^ plots and topology diagrams.

Links are given to PostScript and PDF versions of some of the schematic diagrams. In the case of the LIGPLOT diagram (Figure 2d), a link will load the diagram into the LigPlot^+^ program,^18^ if installed, where it can be further edited interactively and then plotted, if required.

### 3.1 Required accessory programs

As mentioned above, PDBsum*1* requires some external applications to be downloaded and installed, if not already on your system. They are as follows.

- PyMOL—used for generating various ray‐traced 3D images of the protein's structure. Free versions of this program are available for academic users.
- ImageMagick convert—this free utility is used by PDBsum*1* for converting images between one format and another.
- wget—a tool for downloading files from the web, used by PDBsum*1* to retrieve a protein's UniProt data and Pfam domains, where relevant.
- ps2pdf—a utility for converting PostScript files to PDF. It is available as part of the Ghostscript package.

Additional programs that are recommended, but not crucial, are RasMol and LigPlot^+^.

### 3.2 Availability

The PDBsum*1* programs are freely available at https://www.ebi.ac.uk/thornton-srv/software/PDBsum1.

## AUTHOR CONTRIBUTIONS

**Roman A. Laskowski:** Conceptualization (lead); methodology (lead); software (lead); writing – original draft (lead).

## CONFLICT OF INTEREST

The authors declare no potential conflict of interest.

## Data Availability

The data that support the findings of this study are openly available in the Protein Data Bank at https://www.wwpdb.org.^2^

## References

[1] Laskowski RA , Hutchinson EG , Michie AD , Wallace AC , Jones ML , Thornton JM . PDBsum: A web‐based database of summaries and analyses of all PDB structures. Trends Biochem Sci. 1997;22(12):488–490. 9433130 10.1016/s0968-0004(97)01140-7

[2] wwPDB Consortium . Protein Data Bank: The single global archive for 3D macromolecular structure data. Nucleic Acids Res. 2019;47(D1):D520–D528. 30357364 10.1093/nar/gky949 PMC6324056

[3] Milburn D , Laskowski RA , Thornton JM . Sequences annotated by structure: A tool to facilitate the use of structural information in sequence analysis. Protein Eng. 1998;11(10):855–859. 9862203 10.1093/protein/11.10.855

[4] Laskowski RA , Watson JD , Thornton JM . ProFunc: A server for predicting protein function from 3D structure. Nucleic Acids Res. 2005;33(Web Server issue):W89–W93. 15980588 10.1093/nar/gki414 PMC1160175

[5] Laskowski RA , Stephenson JD , Sillitoe I , Orengo CA , Thornton JM . VarSite: Disease variants and protein structure. Protein Sci. 2020;29(1):111–119. 31606900 10.1002/pro.3746 PMC6933866

[6] Laskowski RA , Thornton JM . PDBsum extras: SARS‐CoV‐2 and AlphaFold models. Protein Sci. 2022;31(1):283–289. 34779073 10.1002/pro.4238 PMC8662102

[7] McDonald IK , Thornton JM . Satisfying hydrogen bonding potential in proteins. J Mol Biol. 1994;238(5):777–793. 8182748 10.1006/jmbi.1994.1334

[8] Wallace AC , Laskowski RA , Thornton JM . LIGPLOT: A program to generate schematic diagrams of protein‐ligand interactions. Protein Eng. 1995;8(2):127–134. 7630882 10.1093/protein/8.2.127

[9] Luscombe NM , Laskowski RA , Thornton JM . NUCPLOT: A program to generate schematic diagrams of protein‐nucleic acid interactions. Nucleic Acids Res. 1997;25(24):4940–4945. 9396800 10.1093/nar/25.24.4940 PMC147160

[10] Laskowski RA , MacArthur MW , Moss DS , Thornton JM . PROCHECK ‐ A program to check the stereochemical quality of protein structures. J Appl Cryst. 1993;26:283–291.

[11] Hutchinson EG , Thornton JM . PROMOTIF ‐ A program to identify and analyze structural motifs in proteins. Protein Sci. 1996;5(2):212–220. 8745398 10.1002/pro.5560050204 PMC2143354

[12] Laskowski RA . SURFNET: A program for visualizing molecular surfaces, cavities, and intermolecular interactions. J Mol Graph. 1995;13(5):323–330. 307‐328. 8603061 10.1016/0263-7855(95)00073-9

[13] Sayle RA , Milner‐White EJ . RasMol: Biomolecular graphics for all. Trends Biochem Sci. 1995;20(9):374–376. 7482707 10.1016/s0968-0004(00)89080-5

[14] DeLano WL . The PyMOL molecular graphics system. Palo Alto, CA: DeLano Scientific, 2002.

[15] Oakley AJ , Lo Bello M , Nuccetelli M , Mazzetti AP , Parker MW . The ligandin (non‐substrate) binding site of human Pi class glutathione transferase is located in the electrophile binding site (H‐site). J Mol Biol. 1999;291(4):913–926. 10452896 10.1006/jmbi.1999.3029

[16] Rego N , Koes D . 3Dmol.js: Molecular visualization with WebGL. Bioinformatics. 2015;31(8):1322–1324. 25505090 10.1093/bioinformatics/btu829 PMC4393526

[17] Hutchinson EG , Thornton JM . HERA—A program to draw schematic diagrams of protein secondary structures. Proteins. 1990;8(3):203–212. 2281084 10.1002/prot.340080303

[18] Laskowski RA , Swindells MB . LigPlot+: Multiple ligand‐protein interaction diagrams for drug discovery. J Chem Inf Model. 2011;51(10):2778–2786. 21919503 10.1021/ci200227u

